# Distribution and genotype-phenotype correlation of *GDAP1* mutations in Spain

**DOI:** 10.1038/s41598-017-06894-6

**Published:** 2017-07-27

**Authors:** Rafael Sivera, Marina Frasquet, Vincenzo Lupo, Tania García-Sobrino, Patricia Blanco-Arias, Julio Pardo, Roberto Fernández-Torrón, Adolfo López de Munain, Celedonio Márquez-Infante, Liliana Villarreal, Pilar Carbonell, Ricard Rojas-García, Sonia Segovia, Isabel Illa, Anna Lia Frongia, Andrés Nascimento, Carlos Ortez, María del Mar García-Romero, Samuel Ignacio Pascual, Ana Lara Pelayo-Negro, José Berciano, Antonio Guerrero, Carlos Casasnovas, Ana Camacho, Jesús Esteban, María José Chumillas, Marisa Barreiro, Carmen Díaz, Francesc Palau, Juan Jesús Vílchez, Carmen Espinós, Teresa Sevilla

**Affiliations:** 1 Department of Neurology, Hospital Francesc de Borja, Gandía, Spain; 2 Department of Neurology, Hospital Universitari i Politécnic La Fe, Valencia, Spain; 3 Neuromuscular Research Unit, Instituto de Investigación Sanitaria la Fe (IIS La Fe), Valencia, Spain; 4 Unit of Genetics and Genomics of Neuromuscular and Neurodegenerative Disorders and Service of Genomics and Traslational Geneticis, Centro de Investigación Príncipe Felipe (CIPF), Valencia, Spain; 5 Department of Neurology, Hospital Clínico, Santiago de Compostela, Spain; 6 Neurogenetics Research Group, Instituto de Investigaciones Sanitarias (IDIS), Santiago de Compostela, Spain; 7 Fundación Pública Galega de Medicina Xenómica, Santiago de Compostela, Spain; 8 Centro de Investigación Biomédica en Red de Enfermedades Raras (CIBERER), Intituto Carlos III, Ministry of Economy and Competitiviness, Madrid, Spain; 9 Neuromuscular Disorders Unit, Neurology Department, Hospital Donostia, San Sebastián, Spain; 10 The John Walton Muscular Dystrophy Research Centre, Institute of Genetic Medicine, Newcastle University, Newcastle upon Tyne, UK; 11 Neuroscience Area, Biodonostia Health Research Institute, San Sebastián, Spain; 12 Center for Biomedical Research in the Neurodegenerative Diseases (CIBERNED) Network, Instituto Carlos III, Ministry of Economy and Competitiviness, Madrid, Spain; 13 Department of Neurosciences, School of Medicine, University of the Basque Country (EHU-UPV), San Sebastián, Spain; 14 Department of Neurology and Neurophysiology, Hospital Universitario Virgen del Rocío, Sevilla, Spain; 15 Neuromuscular Diseases Unit, Department of Neurology, Hospital de la Santa Creu i Sant Pau, Universitat Autònoma de Barcelona, Barcelona, Spain; 16 Neuromuscular Unit, Neuropaediatrics Department, Hospital Sant Joan de Déu, Fundacion Sant Joan de Deu, Barcelona, Spain; 17 Neuropaediatrics Department, Hospital la Paz, Madrid, Spain; 18 Department of Pediatrics, Universidad Autónoma de Madrid, Madrid, Spain; 19 Department of Neurology, University Hospital “Marqués de Valdecilla (IDIVAL)”, Santander, Spain; 20 University of Cantabria (UC), Santander, Spain; 21 Neuromuscular Diseases Unit, Department of Neurology, Hospital Clínico San Carlos, Madrid, Spain; 22 Neuromuscular Diseases Unit, Department of Neurology, Hospital Universitari de Bellvitge – IDIBELL, Barcelona, Spain; 23 Child Neurology Unit, Department of Neurology, Hospital Universitario 12 de Octubre, Madrid, Spain; 24 Facultad de Medicina, Universidad Complutense, Madrid, Spain; 25 Department of Neurology, Hospital Universitario 12 de Octubre, Madrid, Spain; 26 Department of Neurology, Hospital Ruber Internacional, Madrid, Spain; 27 Department of Neurophysiology, Hospital Universitari I Politécnic La Fe, Valencia, Spain; 28 Department of Neurology, Hospital General de Alicante, Alicante, Spain; 29 Institut de Recerca Sant Joan de Déu and Hospital Sant Joan de Déu, Barcelona, Spain; 30 Hospital Clínic, Barcelona, Spain; 31 Division of Pediatrics, University of Barcelona School of Medicine and Health Sciences, Barcelona, Spain; 32 Department of Medicine, University of Valencia, Valencia, Spain

## Abstract

Mutations in the *GDAP1* gene can cause Charcot-Marie-Tooth disease. These mutations are quite rare in most Western countries but not so in certain regions of Spain or other Mediterranean countries. This cross-sectional retrospective multicenter study analyzed the clinical and genetic characteristics of patients with *GDAP1* mutations across Spain. 99 patients were identified, which were distributed across most of Spain, but especially in the Northwest and Mediterranean regions. The most common genotypes were p.R120W (in 81% of patients with autosomal dominant inheritance) and p.Q163X (in 73% of autosomal recessive patients). Patients with recessively inherited mutations had a more severe phenotype, and certain clinical features, like dysphonia or respiratory dysfunction, were exclusively detected in this group. Dominantly inherited mutations had prominent clinical variability regarding severity, including 29% of patients who were asymptomatic. There were minor clinical differences between patients harboring specific mutations but not when grouped according to localization or type of mutation. This is the largest clinical series to date of patients with *GDAP1* mutations, and it contributes to define the genetic distribution and genotype-phenotype correlation in this rare form of CMT.

## Introduction

Mutations in the ganglioside-induced differentiation-associated protein 1 (*GDAP1*) gene cause different forms of Charcot–Marie–Tooth disease (CMT). Autosomal recessive mutations have been described in patients with axonal, intermediate and demyelinating forms of the disease, while dominantly inherited mutations cause axonal CMT^1–4^. These mutations are quite rare in Western countries, accounting for less than 1% of the genetically defined CMT patients in most clinical series^5, 6^. In contrast, in certain regions of Spain and Italy, these mutations are the most frequent cause of axonal CMT, accounting for up to 10% of the genetic diagnosis in CMT^7–9^.

The *GDAP1* gene encodes a protein belonging to a glutathione S-transferase (GST) enzyme subfamily in chromosome 8q21.1^10^. GDAP1 is localized in the outer mitochondrial membrane, and is composed of two typical GST domains at the N and C-terminal regions, two alpha loops, a single transmembrane domain, and a hydrophobic domain. Although no GST activity has been demonstrated so far, GDAP1 is involved in the regulation of mitochondrial dynamics and calcium homeostasis^11, 12^.

*GDAP1* autosomal recessive (AR) inherited mutations cause a severe, early onset neuropathy often leading to wheelchair-dependency in the second or third decade. Most of these patients develop unilateral or bilateral vocal cord paresis, and diaphragmatic weakness in the latter stages of the disease^13^. It has been suggested that recessive mutations which cause truncating proteins develop a more severe phenotype, while missense mutations may be associated with a slightly milder course^14^.

On the other hand, autosomal dominant (AD) inherited mutations cause a much milder phenotype, characterized by adult onset, predominantly distal involvement, and slow progression, most of these patients remaining ambulant throughout their lives^15, 16^. Certain clinical characteristics, like dysphonia or dysautonomia, have been described in isolated patients, but no clear genotype-phenotype correlation has been established in AD inherited mutations^9, 17^.

The aim of the study is to describe the distribution of patients with *GDAP1* mutations across Spain and to expand the knowledge of the clinical course and the genotype-phenotype correlation.

## Results

We identified 99 patients from 46 different families harboring causative *GDAP1* mutations. There was no sex predominance in the series and ages ranged from 3–79 years. There were three patients in which only genetic information was available. Nerve conduction studies were performed in 75 patients while other ancillary tests were less frequently performed: leg muscle magnetic resonance imaging in 22 cases and sural nerve pathology in 15.

### Genotype

**Figure 1:**
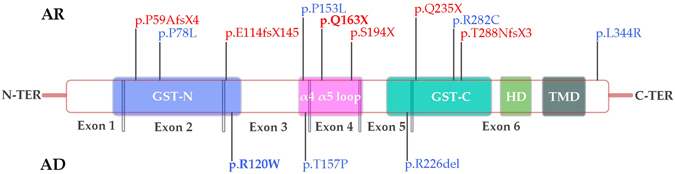
Localization in the *GDAP1* gene of the mutations detected. AD: Autosomal dominant, AR: Autosomal recessive, in blue: missense mutations, in red: truncating mutations.

The genetic information is recorded in Table 1. Thirteen different mutations were detected, 3 inherited in an autosomal dominant fashion and the other 10 were recessive. All had been previously reported. The two most common mutations were AD p.R120W, and AR p.Q163X. The type of mutations was quite diverse including 6 missense, 4 nonsense, 2 frameshift mutations and one splice-site variant. They were localized along exons 2–6 and affected the 2 GST domains and the α-loop predominantly. A schematic diagram of the GDAP1 protein and the mutations found in this series is reproduced in Fig. 1.

**Table 1:** Genotype distribution and effects.

Nucleotide	Amino acid	Effect	Exon	Domain	Patients	Families	Region	Reference
Dominantly inherited								
c.358 C > T	p.R120W	Missense	3	GST-N	47	15	Widespread	Claramunt R, *et al*. ^4^
c.677_679del	p.R226del	Deletion	5	GST-C	10	2	Galicia	García-Sobrino T, *et al*. ^30^
c.469 A	p.T157P	Missense	3	α-loop	1	1	Asturias	Claramunt R, *et al*.^4^
Recessively inherited								
c.487 C > T/c.487 C > T	p.Q163X/p.Q163X	Nonsense	4	α-loop	22	15	North of Spain, Basque region	Cuesta A, *et al*.^1^
c.487 C > T/c.863insA	p.Q163X/p.T288NfsX3	Nonsense/Frameshift STOP	4/6	α-loop	2	1	Valencia	Cuesta A, *et al*. ^1^
c.487 C > T/c.1031 T > G	p.Q163X/p.L344R	Nonsense/Missense	4/6	α-loop	1	1	Cadiz	Sivera R, *et al*.^7^
c.487 C > T/c.581 C > G	p.Q163X/p.S194X	Nonsense	4/5	α-loop	4	2	Valencia, Asturias	Cuesta A, *et al*.^1^
c.487 C > T/c.342_345del	p.Q163X/p.E114fsX145	Nonsense	4/3	α-loop /GST-N	1	1	País Vasco	Claramunt R, *et al*.^4^
c.581 C > G /c.863insA	p.S194X/p.T288NfsX3	Nonsense/Frameshift STOP	5/6	α-loop	2	2	Valencia	Claramunt R, *et al*.^4^
c.172_173delinsTTA/c.311-1 G > A	p.P59AfsX4/splicing variant	Frameshift STOP/No effect	2/int 2	GST-N	1	1	Alicante	Sevilla T, *et al*. 2008^13^
c.844 C > T/c.844 C > T	p.R282C/p.R282C	Missense	6	GST-C	2	1	León	Nelis E, *et al*.^31^
c.863insA/c.863insA	p.T288fsX3/p.T288fsX3	Frameshift STOP	6	α-loop	1	1	Valencia	Cuesta A, *et al*. 2002^1^
c.703 C > G/c.703 C > G	p.Q235X/p.Q235X	Nonsense	6	GST-C	1	1	Baleares	Ortez C, *et al*. ^32^
c.458 C > T/c.458 C > T	p.P153L/p.P153L	Missense	3	α-loop	2	1	Ávila	Kabzinska D, *et al*.^33^
c.233 C > T/c.233 C > T	p.P78Lp.P78L	Missense	2	GST-N	2	1	Madrid/Morocco	Bouhuche A, *et al*.^34^

GST-N: glutathione S-transferase domain in the N-terminal region, GST-C: glutathione S-transferase domain in the C-terminal region.

**Figure 2:**
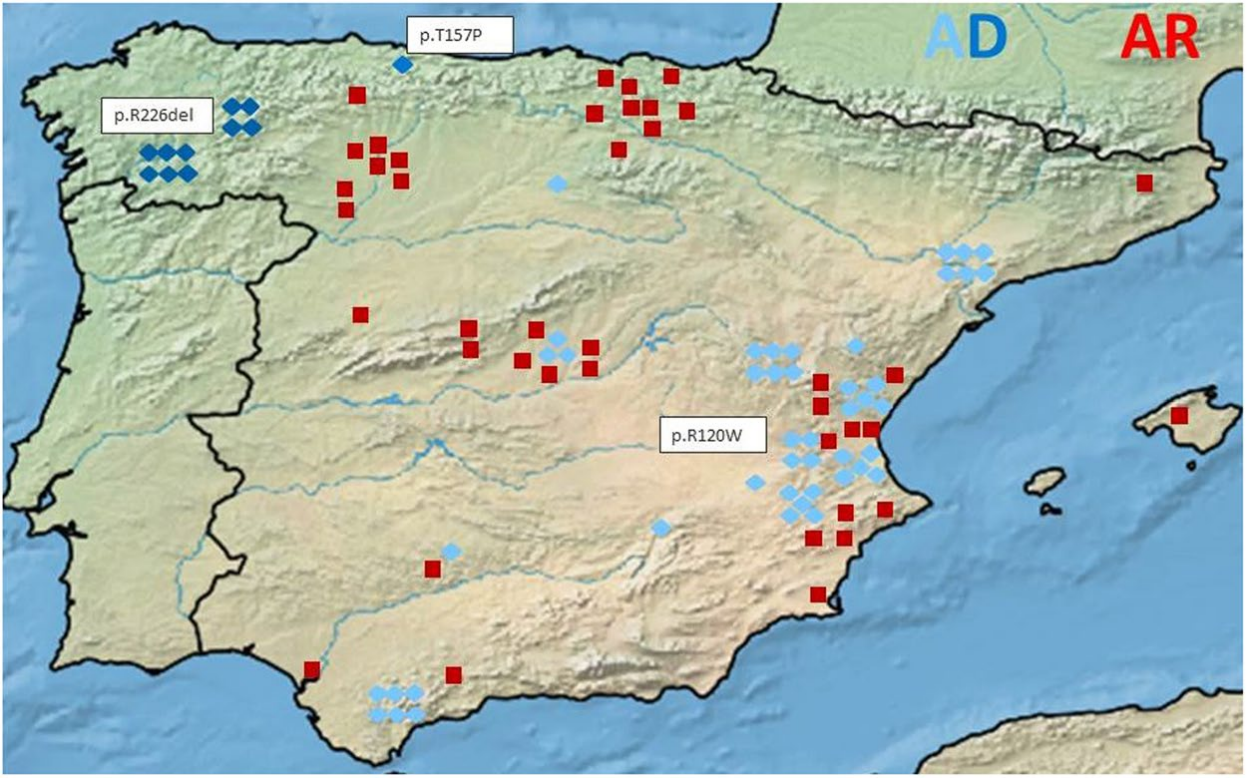
Patient distribution throughout Spain AD: Autosomal dominant, AR: Autosomal recessive, light blue diamond: patient with the AD p.R120W mutation, dark blue diamond: patient with the AD p.R226del mutation, medium blue diamond: patient with the AD p.T157P mutation, red square: patient with AR mutations. The map was created with SimpleMappr, an online tool to produce publication-quality point maps. [Retrieved from http://www.simplemappr.net. May 24, 2017]; Shorthouse, David P. 2010.

The AD p.R120W was detected predominantly in families near the Mediterranean coast, while p.R226del was found only in two families in the region of Galicia, in the Northwest of Spain (Fig. 2). The AR patients were distributed throughout Spain, but there existed a cluster of affected individuals in the North of Spain, especially in the Basque country and neighboring regions.

### Clinical characteristics

The clinical features of the series are summarized in Table 2. Patients were separated between AD and AR inherited mutations because all the severity scores and certain clinical features were different between the groups.

**Table 2:** Clinical characteristics.

	AD inheritance	AR inheritance	Total
n	58	41	99
families	18	28	46
Sex (M/F)	25/33	24/17	49/50
Age at 1rst visit	42.1 yrs (8–79)	27.2 yrs (3–54)	36.4 yrs
Follow up^*^	7 yrs	9.9 yrs	8.4 yrs
Age of independent walking^*^	12 months	15.2 months	13.3 months
Age of onset^*^ (range)	23.8 yrs (4–65)	2.5 yrs (0–12)	13.4 yrs
Asymptomatic (%)	16 (28.6%)	0 (0%)	16 (16.7%)
Sensory symptoms	32 (57.1%)	30 (76.9%)	62 (65.3%)
Motor symptoms	36 (64.3%)	39 (100%)	75 (78.9%)
Autonomic symptoms	0	7 (18.4%)	7 (7.4%)
CMTNS^*^	7.3 (0–26)	22 (8–32)	13.6
CMTES^*^	4.6 (0–21)	15.8 (4–23)	8.9
FDS^*^	1.3 (0–6)	5.2 (2–7)	2.8
Wheelchair-bound (%)	0	30 (75%)	30 (30.9%)
Age wheelchair (yrs)	NA	15,1 (7–52)	15,1 (7–52)
Distal deformities (%)	46 (85.2%)	35 (92.1%)	81 (88%)
Dysphonia (%)	0	22 (56.4%)	22 (23.7%)
Respiratory failure (%)	0	21 (52.5%)	21 (21.2%)
Scoliosis (%)	1 (1.9%)	22 (56.4%)	23 (23.7%)
Death (%)	3 (5.2%)	6 (14.6%)	9 (10%)
Age of death^*^ (yrs)	72 (64–82)	55.7 (42–71)	61.1 (42–82)

*Mean values, AD: Autosomal dominant, AR: Autosomal recessive, yrs: years, CMTNS: Charcot-Marie-Tooth neuropathy score, CMTES: Charcot-Marie-Tooth examination score, FDS: functional Disability Scale, yrs: years, NA: Not applicable. For the % values only the patients with available information were included.

**Figure 3:**
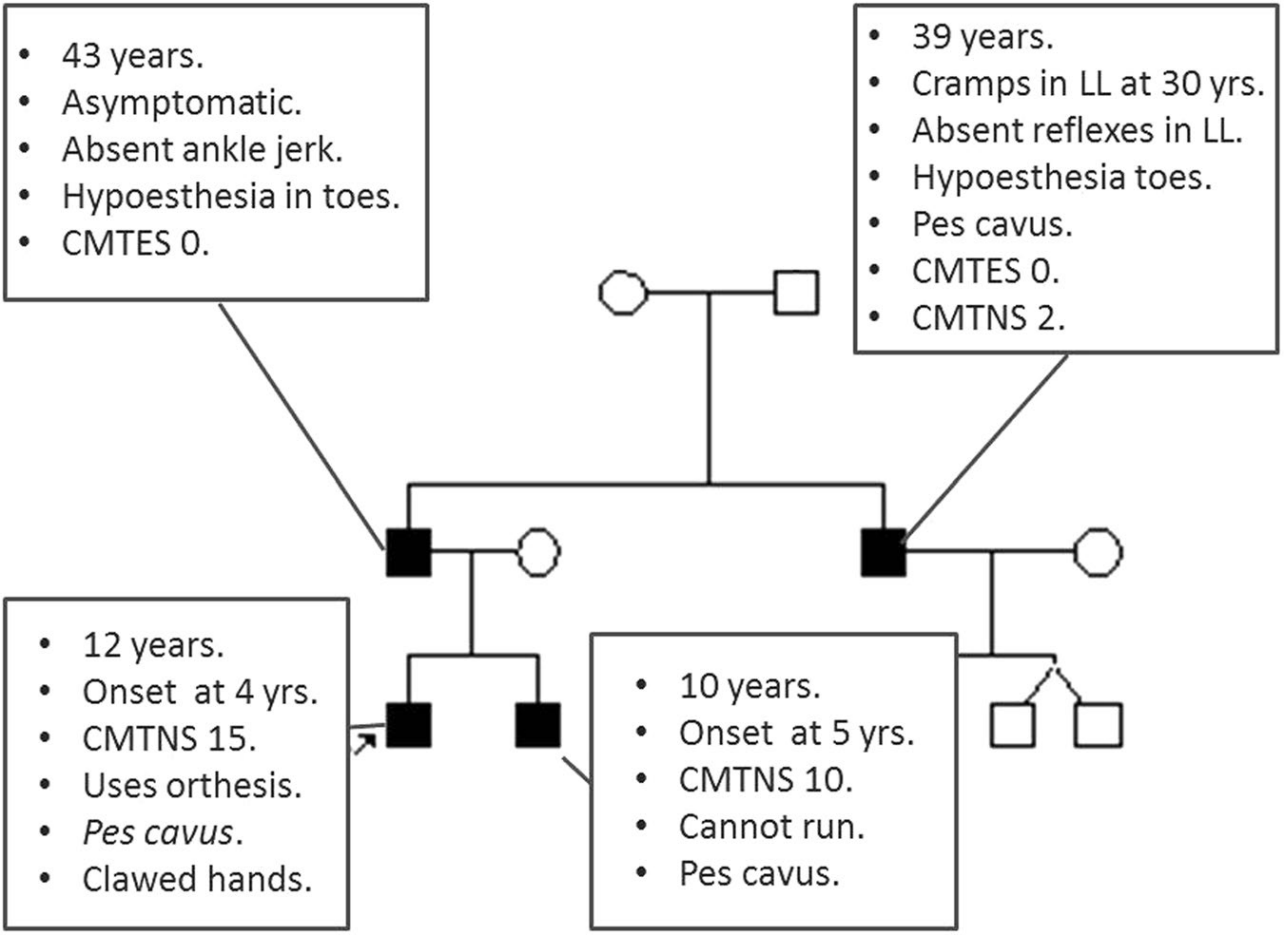
Clinical variability in a family harboring the AD p.R120W mutation. yrs: years, LL: Lower limbs, CMTNS: Charcot-Marie-Tooth neuropathy score, CMTES: Charcot-Marie-Tooth examination score.

Patients with AD inherited mutations had a mild-moderate neuropathy with variable age of onset (4–65 years). The first symptoms most commonly reported were distal lower leg weakness and cramps (83%). Foot deformities (*pes cavus*, Achilles tendon contracture or hammer toes) were quite common (85.2%), but only one patient presented scoliosis and none dysphonia or respiratory failure. Severity scores were usually between the mild-moderate range, and 28.6% considered themselves asymptomatic. There was important clinical variability regarding severity scores even between family members as can be seen in the pedigree of Fig. 3. Three patients died during follow-up, but the cause of death was not related with the neuropathy.

Patients with AR inherited mutations suffered a severe early onset neuropathy (0–12 years) causing a great disability. Most of the patients were wheelchair-bound before age 20, and only three patients older than 40 years remained ambulant. Dysphonia was present in more than half of the AR patients and appeared usually in the second decade (9–35 years). Respiratory dysfunction was also quite common, and 19.5% of the patients required non-invasive ventilation (29–47 years). Intra-familiar variability was not prominent, although most were sporadic patients. Six patients died, two because of cardiovascular events, one of hepatic cirrhosis, and three due to infections that could be related to the severe disability inherent to the disease course.

### Nerve conduction studies

The electrophysiological findings are detailed in Table 3. In AD patients there was a decrease of CMAP and SNAP amplitudes with normal conduction velocities, corresponding to a mild axonal motor and sensory neuropathy. Of the 12 clinically asymptomatic patients that underwent nerve conduction studies, in 8 we detected a decrease in SNAP of the sural nerve and/or CMAP of the peroneal nerve. The other 4 asymptomatic patients had normal nerve conduction studies, but 3 of them had detectable abnormalities in needle electromyography (large polyphasic motor unit action potentials in distal lower limb muscles with no spontaneous muscular activity), while it was not performed in the other patient.

**Table 3:** Motor and sensory nerve conduction studies.

	AD inheritance	AR inheritance	Total
n	48	28	76
Age NCS^*^	38.5 yrs (8–82)	20.2 yrs (2–48)	30.8 yrs
Disease course NCS^*^	18 yrs	17.3 yrs	17.6 yrs
Ulnar CMAP^*^	10.7 mV (0.9–20.6)	3.8 mV (0.1–9.1)	9.5 mV
Ulnar MCV^*^	58.4 m/s (47.3–68)	43.9 m/s (42–57.6)	55.4 m/s
% Unexcitable	0/27 (0%)	12/19 (63.2%)	12/46 (26.1%)
Median CMAP^*^	9.7 mV (3.2–23.4)	2 mV (0.4–11.7)	7.6 mV
Median MCV^*^	56.6 m/s (48–69.1)	42 m/s (30.4–66.7)	53.4 m/s
% Unexcitable	0/39 (0%)	11/23 (47.8%)	11/64 (17.2%)
Peroneal CMAP^*^	4.8 mV (0.3–11.9)	0.5 mV (0.3–0.6)	4.5 mV
Peroneal MCV^*^	44.8 m/s (38–61)	40.1 m/s (40.1)	44.7 m/s
% Unexcitable	7/47 (14.9%)	18/20 (90%)	25/67 (37.3%)
Ulnar SNAP^*^	10.7 μV (1.4–27)	2.1 μV (0.2–6.1)	8.5 μV
Ulnar SCV^*^	48.4 m/s (34.7–64.7)	46.1 m/s (34.3–55.7)	47.8 m/s
% Unexcitable	2/22 (9.1%)	8/14 (57.1%)	10/36 (27.8%)
Median SNAP ^*^	11.4 μV (1.6–32.6)	3.5 μV (0.2–16)	9.2 μV
Median MCV^*^	47.7 m/s (36.3–65.1)	40.8 m/s (30.4–53)	45.7 m/s
% Unexcitable	4/39 (10.3%)	14/26 (53.8%)	18/65 (27.7%)
Sural SNAP^*^	5.5 μV (0.7–13.9)	4,8 μV (2.6–7.2)	5.5 μV
Sural SCV^*^	43.1 m/s (29–65.9)	46.8 m /s (43.9–49.6)	43.4 m /s
% Unexcitable	18/46 (39.1%)	19/22 (86.4%)	37/68 (54.4%)

*Mean values, AD: Autosomal dominant, AR: Autosomal recessive, NCS: Nerve conduction studies, CMAP: Compound muscle action potential, MCV: motor conduction velocity, SNAP: Sensory nerve action potential, SCV: sensory conduction velocity, yrs: years, mV: millivolts, μV: microvolts. For the % values only the patients with available information were included.

**Figure 4:**
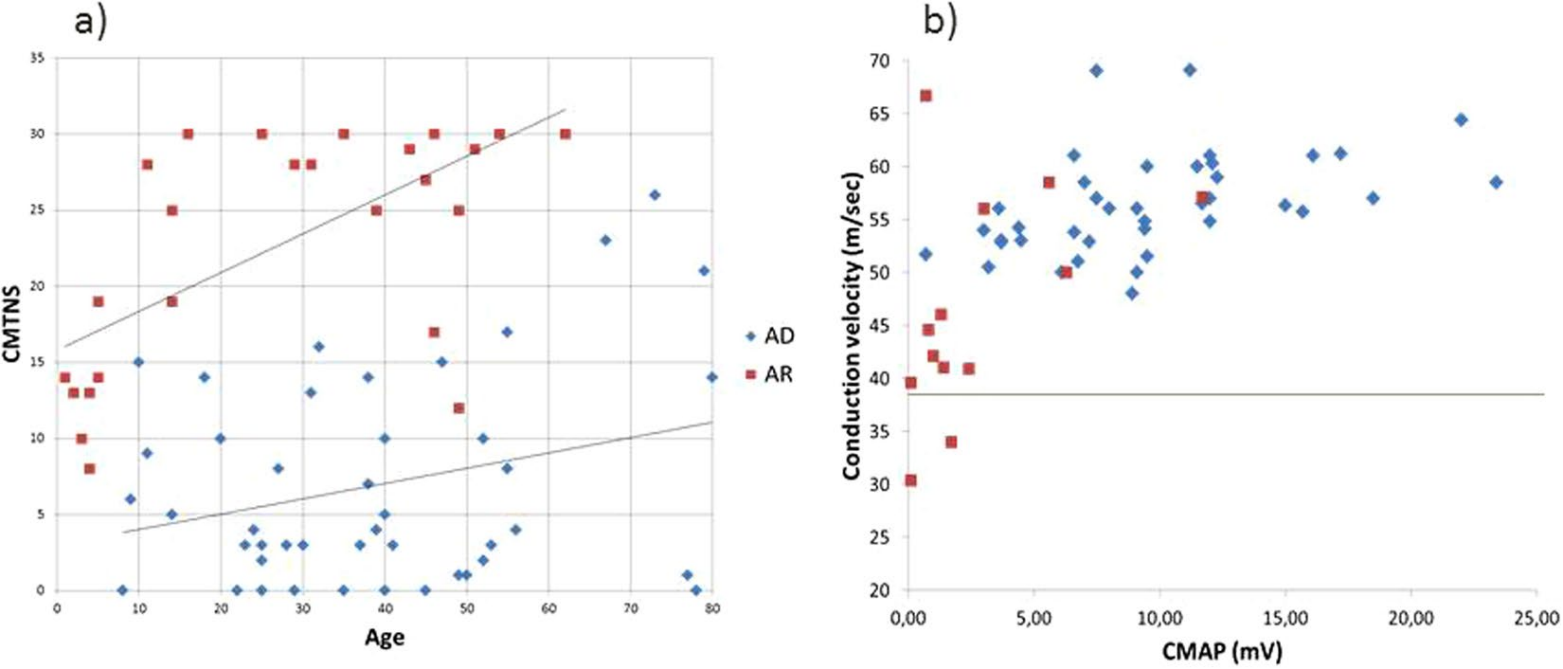
(**a**) Dispersion chart of the CMTNS scores and ages in the first examination of patients with AD and AR inherited mutations. (**b**) Dispersion chart of the CMAP of the median nerve the motor conduction velocity in patients with AD and AR inherited mutations. AD: Autosomal dominant, AR: Autosomal recessive, CMTNS: Charcot-Marie-Tooth neuropathy score, CMAP: Compound muscle action potential.

In AR patients the decrease in CMAP and SNAP was much more marked, as were the number of nerves that were unexcitable when explored. These findings were more severe in the lower limb nerves; in fact all patients over 25 years had unexcitable sural and peroneal nerves. Conduction velocities were generally not reduced except in nerves with very low amplitudes (Fig. 4b). In these cases, when conduction velocity was measured to proximal muscles, it was always within the normal range.

### Muscle magnetic resonance imaging

Lower limb muscle magnetic resonance imaging findings are detailed in Table 4, and representative images are shown in the supplementary Figure S1. It was performed in 22 patients, 19 of them harboring the AD p.R120W mutation. In those patients the findings were in concordance with that previously reported including fatty infiltration with a distal to proximal gradient^16^. In 3/5 clinically asymptomatic patients, there was detectable fatty substitution in the intrinsic foot muscles and in 2/5 also in the muscles of the calf. In the 2 asymptomatic patients with normal muscle MRI, the muscles of the feet were not studied. In the calf, the muscles of the posterior compartment (soleus > gastrocnemius) were affected earlier and more severely than those of the anterolateral compartment.

**Table 4:** Muscle magnetic resonance imaging of the lower limbs.

Genotype	Age	Disease course (years)	CMTNS	IFM	Soleus	Gastrocnemius	DPC	AC	LC	Thigh
p R120W	71	A	0*	2	2	2	4	4	4	NP
p R120W	51	A	1*	NP	0	0	0	0	0	NP
p R120W	50	5	2*	NP	0	0	0	0	0	NP
p R120W	23	3	3	3	2	3	0	0	1	NP
p R120W	37	A	3	3	0	0	0	0	0	NP
p R120W	49	10	4*	3	2	2	0	0	0	NP
p R120W	56	A	4	NP	0	0	0	0	0	NP
p R120W	52	34	4*	4	4	4	1	2	2	NP
p R120W	40	A	5	2	1	1	0	0	0	NP
p R120W	38	6	7	3	1	2	0	0	1	NP
p R120W	27	21	8	NP	4	4	0	1	0	1
p R120W	68	18	9	4	4	4	2	3	3	1
p R120W	24	12	10	2	1	1	0	0	0	NP
p R120W	20	6	10	4	4	4	2	3	3	3
p R120W	32	23	11*	4	3	4	0	1	1	NP
p R120W	58	43	11*	4	4	3	3	1	2	2
p.R120W	31	18	13	4	3	3	2	2	3	NP
p R120W	55	50	17	4	4	4	4	4	4	3
p R120W	73	48	26	4	4	4	3	4	3	3
p.R226del	55	50	8	3	4	3	3	2	2	2
p.R226del	45	A	2	NP	0	0	0	0	0	0
p.Q163X/p.L344R	49	37	12	4	4	4	4	3	3	3

CMTNS: Charcot-Marie-Tooth neuropathy score, IFM: Intrinsic foot muscles, DPC: Deep posterior compartment of the calf, AC: Anterior compartment of the calf, LC: Lateral compartment of the calf, A: Asymptomatic, *CMTES score values, NP: Not performed.

MRI was performed in two patients with the AD p.R226del mutation. In one symptomatic patient there were detectable abnormalities consisting in fatty infiltration with a distal to proximal gradient, and the same posterior > anterolateral pattern in the calf. The other was asymptomatic, although minor abnormalities could be detected in the clinical examination. The muscle MRI in this patient was normal in the calf and thigh muscles, but the feet were not studied.

One patient with an AR p.Q163X/p.L344R mutation also underwent muscle MRI testing. He was the AR patient with the less severe phenotype (CMT neuropathy score - CMTNS: 12, Functional disability score - FDS: 2, age 49 at the time of the MRI) and there was complete fatty substitution of the muscles in the feet and posterior compartment of the calf, but in the anterolateral muscles of the calf and the thigh muscles, fatty infiltration was only partial.

### Nerve pathology

Sural nerve biopsy was performed in 15 patients, 8 with AR and 7 with AD inherited mutations. Representative images are shown in the supplementary Figure S1. The pathologic characteristics of 7 of these patients had been previously been reported, and are similar to the other 8 patients^1, 16, 18^. The main findings were concordant with an axonal neuropathy (depletion of myelinated fibers, myelin thinning, and rather frequent regenerative clusters) with minor myelin abnormalities (abnormal myelin foldings and occasional onion bulb-like formations). The fiber loss was especially prominent in AR patients.

### Genotype-phenotype correlation

The most important genetic factor that influenced the phenotype was the mode of inheritance. Certain clinical features like the presence of dysphonia and respiratory dysfunction were only present in patients with AR inheritance and scoliosis was more frequent in patients with AR inheritance (p < 0.001) (Table 2). In this group, 75% of the patients were wheelchair-bound at a median age of 15 years. By contrast none in the group of AD inheritance lost ambulation with a median follow-up of nearly 7 years. The presence of any kind of neuropathic symptoms was more frequent in patients with AR inheritance, as were higher severity scores in any of the scales employed (p < 0.05). In Fig. 4a the CMTNS scores of the first examination in both groups can be compared. With a logistic regression model, the only co-variables that could independently predict a higher CMTNS score were the mode of inheritance: OR 38, CI 95% [4–474.2] and the disease duration OR 1.1 CI 95% [1.05, 1.19].

Regarding the patients with AD inheritance, we detected minor clinical differences between the two most frequent mutations. The members of the two families with the p.R226del mutation had a slightly milder disease course. Also, in the p.R120W mutation ankle extensors and flexors were affected to a similar degree (3.9 and 3.7 mean MRC scores respectively) while in those with the p.R226del mutation weakness was more marked in ankle extension (4.3 and 4.7 mean MRC scores).

In the patients with AR inherited mutations no clinical differences could be detected when grouping patients according to localization of the mutation or mutation type. Neither could we detect any statistically significant difference in age of onset, years to wheelchair, or other severity scores between patients with ‘truncating’ mutations and those with two missense mutations. In patients with the same mutation, clinical variability regarding severity was not prominent. Regarding specific mutations, the only two patients with the homozygous p.R282C mutation seemed to have a milder clinical course, as did the only patient compound heterozygous for the p.Q163X and p.L344R mutations.

## Discussion

This multicentric cross-sectional study provides information about 99 patients with CMT caused by *GDAP1* mutations. In Spain the relative frequency of *GDAP1* mutations has been reported to be as high as 13% of the genetically defined CMT in certain regions^7^. Patients in this series were distributed throughout Spain, and in most centers comprised the first cause of CMT2, excluding *GJB1* mutations. This contrasts with the scarcity of these mutations in most Western Countries, accounting for less than 2% of the genetically defined patients in series from the United Kingdom, Germany, United States, and also in the cohort of patients from the Inherited Neuropathies Consortium^5, 6, 19, 20^. On the other hand, there have been reports of regional Italian clinical CMT series that found frequencies of *GDAP1* mutations of > 7%, and after analyzing a group of patients referred to the Medical Genetics Unit of the University of Genoa, the authors conclude that *GDAP1* mutations should be the first genetic diagnosis to be considered in an Italian patient with CMT2^8, 9^. Further information about the relative frequency of *GDAP1* mutations in the South of France and other countries of the Mediterranean coastline (Greece, Turkey, Morocco, etc.) will be needed to clarify the geographical distribution of these mutations.

In Spain, the high frequency of *GDAP1* mutations among CMT2 patients is in part due to the high frequency of two mutations. The p.R120W substitution accounted for 81% of AD patients and was detected mostly in the Mediterranean basin, while p.Q163X was detected either as a homozygous or compound heterozygous mutation in 73.2% of AR patients, especially in the North/Northwest of Spain. Both of these mutations had been studied previously with haplotype analysis and postulated as having a founder effect in the region, although they have been detected in families in other European and American countries^1, 16, 21^.

Regarding the phenotype, the most important factor that influences the clinical characteristics of these patients is the mode of inheritance (Table 2). The patients with AR inherited mutations have a more severe course and certain clinical features like dysphonia and respiratory dysfunction were exclusively found in this group^13, 18^. In fact when the CMTNS scores of the series were modeled with a logistic regression analysis, the only factor that could independently predict higher CMTNS scores apart from disease evolution was AR inheritance. Although AR patients had a more severe course as a group, there existed a certain overlap between the severity scores when grouped by the inheritance pattern (Fig. 4a).

For example, there were two AR patients with a milder phenotype than the rest of the cohort (CMTNS scores of 12 and 16 at ages 49 and 46 respectively). The latter is still ambulant with crutches at 56 years of age, and harbors the homozygous missense p.R282C change. This mutation has only been detected in him and his sister, who has a slightly more severe phenotype and became wheelchair-bound only at age 52. The other patient was a compound heterozygous for p.Q163X and p.L344R, and remained ambulant with orthosis until his death due to hepatic cirrhosis at age 55. Even taking into account these outliers, when the clinical characteristics or severity scores were compared according to the localization of the mutation or mutation type, no differences could be established. It has been reported that patients with two ‘truncating’ mutations have a more severe phenotype than those with two missense mutations, but when we compared age of onset, years to wheelchair or other impairment scores we could not replicate these findings^14^.

In patients with AD inheritance, phenotypic variability regarding severity was prominent, even within affected members of the same family. There are three AD patients with a severe neuropathy according to CMTNS, one of them needing a walker for ambulation; while 28.6% considered themselves asymptomatic, two of them older than 70 years. The factors that influence this variability are largely unknown, but the search for genetic modifiers in this disease led to the detection of a concomitant *junctophilin-1* (*JPH1*) change which modified GDAP1 function in one of the families with the p.R120W mutation included in this series^22^. *JPH1* substitutions have been detected in two other families, one of them is represented in Fig. 3 where the moderately affected siblings have inherited a change in *JPH1* from their mother, and the p.R120W *GDAP1* mutation from their practically asymptomatic father (in press). In any case, important clinical variability can be found in at least 6 other families with the p.R120W mutation and no concomitant changes in *JPH1* have been detected.

Minor differences could be established between the phenotype in the p.R226del and p.R120W mutations but these have to be interpreted in the context of the clinical variability inherent to this disease. In the p.R120W mutation ankle flexion weakness could be detected as soon as ankle extension weakness, and fatty substitution predominated in the posterior compartment of the calf, while in the p.R226del mutation the MRI findings were similar in the symptomatic patient, but in the clinical examination ankle extension weakness predominated. In any case the description of more patients will be necessary to confirm these findings.

Autosomal dominant *GDAP1* mutations have been described as having an incomplete penetrance, but in this series all asymptomatic patients had detectable abnormalities in clinical examination or ancillary testing except one. Of the 16 asymptomatic patients 9 had abnormalities in the examination, 11/12 in the electrophysiological studies and 3/6 in the MRI. The only patient that had normal examination and ancillary tests did not undergo needle electromyography or feet MRI, which are the most sensitive tests in this series. This is in keeping with the findings in the Italian p.Arg120Gly mutation where the phenotypic variability mimicked a reduced penetrance, but electrophysiological studies were unequivocally abnormal in affected patients^23^.

These findings enhance the complexity of the clinical phenotype and of predicting the clinical course in *GDAP1* mutations. This last can be especially challenging, but is necessary for the development of future clinical trials. In this regard there are several considerations that are important in this CMT subtype. In AR patients the severity of the phenotype determined that the scales employed could not adequately assess disease progression except in the first or second decade, as afterwards there was a clear ceiling effect in both the CMTNS and FDS scales. In these patients ancillary testing could contribute scarcely as MRI of the lower limbs was affected to a great degree and the rate of change in these patients would be difficult to interpret. In AD patients, apart from the intra-familiar variability, the analysis of the available longitudinal information shows that the change rate of CMTNS per year in one same patient can be quite variable (data not shown). The responsiveness to change probably could be improved if the CMTNS version 2, or the Rasch-wheighted CMTNS version 2 scores had been used, but this was impossible as radial nerve conduction studies were available for only a minority of the patients^24^. In any case, taking into account the rarity of this CMT subtype, and the intra-familiar variability, more objective measures of disease progression will be necessary, like the MRI biomarkers that have already been developed for CMT1A^25^.

This is the largest clinical series of CMT patients with *GDAP1* mutations and it contributes to expand the knowledge of the genetic distribution and genotype-phenotype correlation of this disease.

## Methods

### Subjects

This cross-sectional retrospective observational study included all CMT patients with causative *GDAP1* mutations evaluated at 14 centers throughout Spain during the 2000–2016 timeframe. 74 Patients were selected from a nationwide register of hereditary neuropathies including 1405 patients, 307 of them defined as CMT2. This registry was developed in 2012 being part of the Spanish Registry of Neuromuscular Diseases project (NMD-ES). After a call for patients in the Neuromuscular Work Group of the Spanish Neurology Society (SEN), 25 other patients were identified. All patients included have signed an informed consent form specific for the NMD-ES registries which was approved by the Ethics Committee of the Hospital de la Santa Creu i Sant Pau. Genetic, demographic, clinical, electrophysiological, and pathologic information was evaluated. Only genetically confirmed patients were included. All experiments were performed in accordance with relevant guidelines and regulations.

### Mutational analysis

Mutation analysis of the *GDAP1* gene (NM_018972.2) was performed mostly in the same center as previously described using an Applied Biosystems 3730xl DNA analyzer (Foster City, CA, USA)^1^. To investigate the novelty of the identified variants, the following databases were consulted: dbSNP (http://www.ncbi.nlm.nih.gov/SNP), ESP6500 (http://evs.gs.washington.edu/EVS/), ExAC (http://exac.broadinstitute.org/), and CSVS (http://csvs.babelomics.org/). In silico analysis was performed to predict the pathogenicity of the novel variants, using SIFT (http://sift.bii.a-star.edu.sg/) and PolyPhen-2 (http://genetics.bwh.harvard.edu/pph2/) algorithms for missense changes and NNSPLICE v0.9 (http://www.fruitfly.org/seq_tools/splice.html) for splicing alterations. Whenever possible, segregation analysis was performed.

### Clinical assessments

A standardized history and symptom questionnaire was employed to collect the basal information. Clinical examinations included strength using the standard Medical Research Council (MRC) scale, pinprick and vibratory sensory loss, reflexes, as well as a general and neurologic examination. Severity of the neuropathy was evaluated with the CMT neuropathy score version 1 (CMTNSv1) as a significant proportion of these patients had not undergone the neurophysiologic radial nerve testing which is mandatory for the CMTNS version 2^26, 27^. The CMT examination score (CMTES) was used for patients without nerve conduction studies. The functional disability scale (FDS), which grades the functional impairment from 0–8, was used to measure the disability status^28^. Respiratory dysfunction was defined by standard spirometry values in each center.

### Nerve conduction studies

Motor and sensory nerve conduction velocities (NCVs) were performed using standard techniques. Temperature was controlled during the procedure and kept at more than 32 °C. Compound muscle action potential amplitudes (CMAP), sensory nerve action potential amplitudes (SNAP), conduction velocities and distal latencies were recorded.

### Muscle magnetic resonance imaging (MRI)

MRI was performed on the feet and distal legs. The protocol employed was the same as described previously, and fatty substitution was graded from 0 to 4 as follows: 0, no fat signal in muscle; 1, some fatty streaks; 2, fat occupying a minor part of muscle; 3, similar amount of fat and muscle tissue; 4, fat occupying the greater part of muscle^16, 29^. The muscles in the calf were grouped according to 4 anatomical compartments: anterior compartment (*tibialis anterior*, *extensor hallucis longus*, and *extensor digitorum longus*), lateral compartment (*peronei longus* and *brevis*), superficial posterior compartment (*soleus* and *gastrocnemius*), and deep posterior compartment (*tibialis posterior*, *flexor digitorum longus*, and *flexor hallucis longus*).

### Nerve pathology

Sural nerve biopsy was performed before the genetic diagnosis was confirmed, or for investigational reasons after specific informed consent. Sections were evaluated under a light microscope, and morphometric data was analyzed when available^18^.

### Statistical studies

Patient characteristics, data from clinical examination, electrophysiological, examination and physical disability were analyzed using descriptive statistics with the program R (version 3.3.3), the ordinal package (version 2015.6–28) and brglm (0.5–9). A logistic regression model was developed to determine the co-variables that could predict a higher CMTNS score.

### Data Availability

The datasets generated during and/or analyzed during the current study are available from the corresponding author on reasonable request.

## Electronic supplementary material

Supplementary Information
