# Supplementary material for: Distribution and genotype-phenotype correlation of GDAP1 mutations in Spain
Source: Sci Rep. 2017 Jul 27;7:6677. doi: 10.1038/s41598-017-06894-6 (PMC5532232; doi:10.1038/s41598-017-06894-6)
Supplement: Supplementary file 1 — Supplementary Information [file 41598_2017_6894_MOESM1_ESM.pdf]

Distribution and genotype-phenotype correlation of *GDAP1* mutations in Spain

Rafael Sivera*[1], Marina Frasquet[2,3], Vincenzo Lupo[4], Tania García-Sobrino[5], Patricia Blanco-Arias[6,7,8], Julio Pardo[5], Roberto Fernández-Torrón[9,10,11,12,13], Adolfo López de Munain[9,11,12,13,14], Celedonio Márquez-Infante[15], Liliana Villarreal[15], Pilar Carbonell[15], Ricard Rojas-García[8,16], Sonia Segovia[8], Isabel Illa[8,16], Anna Lia Frongia[17], Andrés Nascimento[8,13,17], Carlos Ortez[8,13,17], María del Mar García-Romero[18], Samuel Ignacio Pascual[18,19], Ana Lara Pelayo-Negro[12,20,21], José Berciano[12,20,21], Antonio Guerrero[22], Carlos Casasnovas[23], Ana Camacho[24,25], Jesús Esteban[26,27], María José Chumillas[28], Marisa Barreiro[3], Carmen Díaz[29], Francesc Palau[8,30,31,32], Juan Jesús Vílchez[2,3,8,33], Carmen Espinós[4], Teresa Sevilla[2,3,8,33].

[1]Department of Neurology, Hospital Francesc de Borja, Gandía, Spain.

[2]Department of Neurology, Hospital Universitari I Politécnic La Fe, Valencia, Spain.

[3]Neuromuscular Research Unit, Instituto de Investigación Sanitaria la Fe (IIS La Fe), Valencia, Spain.

[4]Unit of Genetics and Genomics of Neuromuscular and Neurodegenerative Disorders and Service of Genomics and Traslational Geneticis, Centro de Investigación Príncipe Felipe (CIPF), Valencia, Spain.

[5]Department of Neurology, Hospital Clínico, Santiago de Compostela, Spain.

[6]Neurogenetics Research Group, Instituto de Investigaciones Sanitarias (IDIS), Santiago de Compostela, Spain.

[7]Fundación Pública Galega de Medicina Xenómica, Santiago de Compostela, Spain.

[8]Centro de Investigación Biomédica en Red de Enfermedades Raras (CIBERER), Spain.

[9]Neuromuscular Disorders Unit, Neurology Department, Hospital Donostia, San Sebastián, Spain.

[10]The John Walton Muscular Dystrophy Research Centre, Institute of Genetic Medicine, Newcastle University, Newcastle upon Tyne, UK.

[11]Neuroscience Area, Biodonostia Health Research Institute, San Sebastián, Spain.

[12]Center for Biomedical Research in the Neurodegenerative Diseases (CIBERNED) Network, Spain.

[13]Instituto Carlos III, Ministry of Economy and Competitiveness, Madrid, Spain.

[14]Department of Neurosciences, School of Medicine, University of the Basque Country (EHU-UPV), San Sebastián, Spain.

[15]Department of Neurology and neurophysiology, Hospital Universitario Virgen del Rocío, Sevilla, Spain.

[16]Neuromuscular Diseases Unit, Department of Neurology, Hospital de la Santa Creu i Sant Pau, Universitat Autònoma de Barcelona Barcelona, Spain

[17]Neuromuscular Unit, Neuropaediatrics Department, Hospital Sant Joan de Déu, Fundacion Sant Joan de Deu, Barcelona, Spain.

[18]Neuropaediatrics Department, Hospital la Paz. Madrid, Spain.

[19]Department of Pediatrics. Universidad Autónoma de Madrid, Madrid, Spain

[20]Department of Neurology, University Hospital “Marqués de Valdecilla (IDIVAL)”, Santander, Spain.
[21]University of Cantabria (UC), Santander, Spain.
[22]Neuromuscular Diseases Unit, Department of Neurology, Hospital Clínico San Carlos, Madrid, Spain.
[23]Neuromuscular Diseases Unit, Department of Neurology, Hospital Universitari de Bellvitge – IDIBELL, Barcelona, Spain.
[24]Child Neurology Unit, Department of Neurology, Hospital Universitario 12 de Octubre. Madrid, Spain.
[25]Facultad de Medicina, Universidad Complutense, Madrid, Spain.
[26]Department of Neurology, Hospital Universitario 12 de Octubre. Madrid, Spain.
[27]Department of Neurology, Hospital Ruber Internacional. Madrid, Spain.
[28]Department of Neurophysiology, Hospital Universitari I Politécnic La Fe, Valencia, Spain.
[39]Department of Neurology, Hospital General de Alicante, Alicante, Spain.
[30]Institut de Recerca Sant Joan de Déu and Hospital Sant Joan de Déu, Barcelona, Spain.
[31]Hospital Clínic, Barcelona, Spain.
[32]Division of Pediatrics, University of Barcelona School of Medicine and Health Sciences, Barcelona, Spain.
[33]Department of Medicine, University of Valencia, Valencia, Spain.

*Corresponding author: Department of Neurology, Hospital Francesc de Borja, Av Medicina 6, Gandía, Spain; Tel. 0034-962843500, FAX. 0034-962849700; rafasivera@gmail.com

Supplementary figure S1: Representative MRI and pathology findings in patients with *GDAP1* mutations

A-B: Axial T1-weighted magnetic resonance imaging of the foot and calf muscles in an asymptomatic patient with the AD p.R120W mutation, showing fatty infiltration in the intrinsic foot muscles, and in the soleus and gastrocnemius.

C: Axial T1-weighted magnetic resonance imaging of the calf in a patient harboring the AD p.R226del mutation. The patient has a CMTNS score of 8 and fatty infiltration in all the muscle compartments of the calf, predominantly affecting the posterior compartment.

D: Axial T1-weighted magnetic resonance imaging of the calf in a patient who is compound heterozygous for the p.Q163X and p.L344R mutations, showing prominent fat substitution in all the muscles in the calf .

E: Sural nerve semi-thin transverse section with x40 magnification of a patient with a moderately mild phenotype (CMTNS: 9) due to a p.R120W AD mutation. There is mild myelinated fiber loss with relative preservation of large fibers, abundant regenerative clusters, and few fibers with thin myelin sheaths. The myelin can adopt irregular shapes in some fibers due to the presence of abnormal myelin folding.

F: Sural nerve semi-thin transverse section with x40 magnification of a patient with a severe phenotype due to AR inherited p.Q163X and p.T288NfsX3 mutations. There is a pronounced depletion of myelinated fibers, remaining only the smaller ones. There are also proliferative features corresponding to pseudo onion-bulb formations surrounding thinly myelinated axons or regenerative clusters.

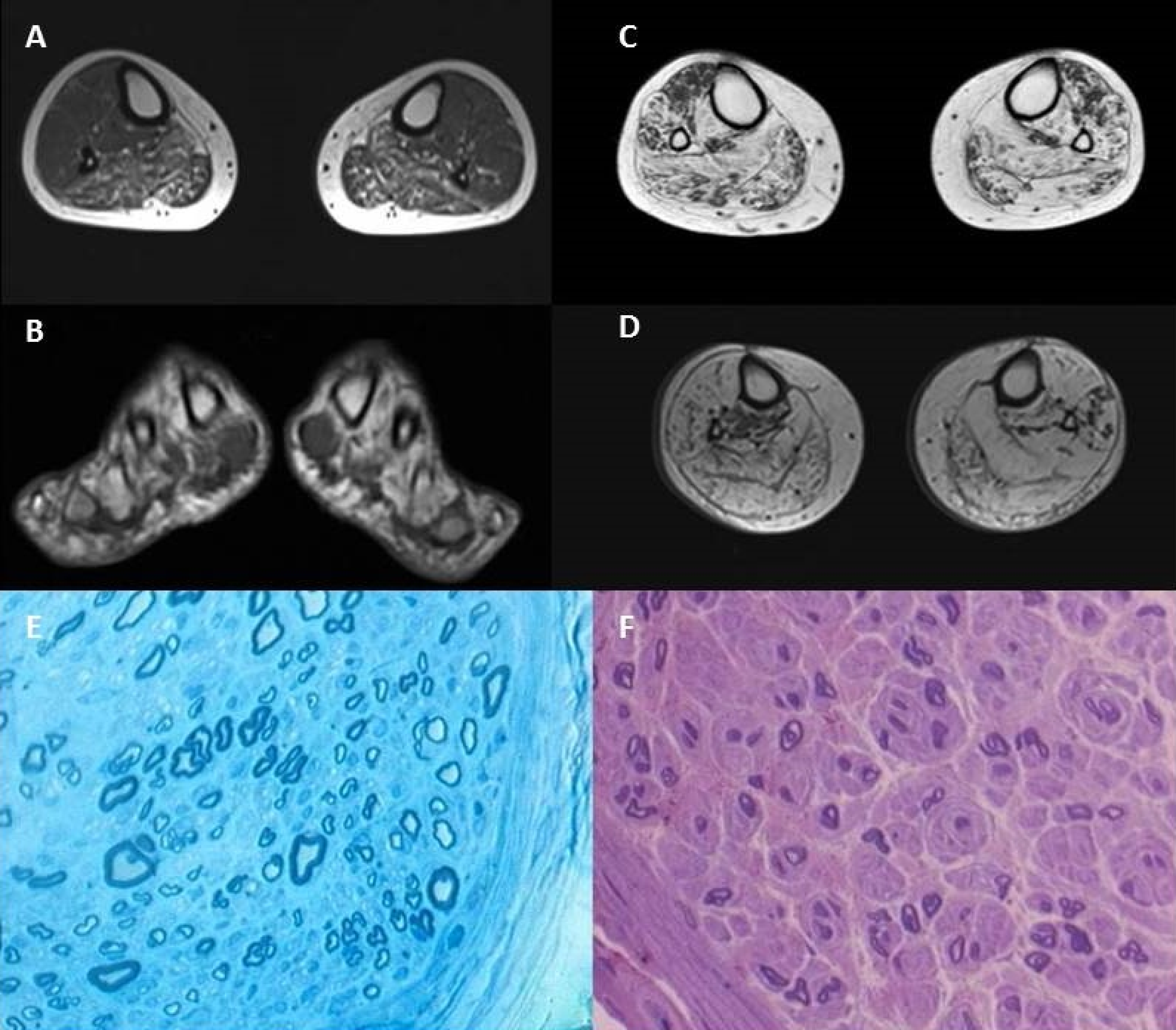
A
B
C
D
E
F
